# The role and mechanism of histone lactylation in health and diseases

**DOI:** 10.3389/fgene.2022.949252

**Published:** 2022-08-23

**Authors:** Yumei Xie, Hongxia Hu, Maoting Liu, Tingting Zhou, Xi Cheng, Wei Huang, Ling Cao

**Affiliations:** ^1^ Department of Nephrology, Sichuan Clinical Research Center for Nephropathy, The Affiliated Hospital of Southwest Medical University, Luzhou, Sichuan, China; ^2^ Department of Endocrinology and Metabolism, Metabolic Vascular Diseases Key Laboratory of Sichuan Province, The Affiliated Hospital of Southwest Medical University, Luzhou, Sichuan, China

**Keywords:** histone lactylation, epigenetic, gene transcription, inflammation, tumor, novel posttranslational modification, lactate

## Abstract

Whether under anaerobic or aerobic conditions, glycolysis results in production of lactate. Increasing evidence suggests that lactate serves as a multifunctional signaling molecule that develops non-metabolic activities in addition to serving as a key metabolite to link glycolysis and oxidative phosphorylation. Histone posttranslational modification patterns (HPTMs) are essential epigenetic processes controlling a variety of biological activities. Proteomics based on mass spectrometry (MS) has been used to progressively reveal new HPTMs. Recent discoveries of histone lactylation modification mediated by lactate and subsequent research demonstrating its involvement in cancer, inflammation, lung fibrosis, and other conditions suggest that it plays a significant role in immune regulation and homeostasis maintenance. This review provides a brief overview of the complicated control of histone lactylation modification in both pathological and physiological conditions.

## Introduction

As part of the glycolysis process, lactate dehydrogenase (LDH) catalyzes a specific type of hydroxycarboxylic acid. It is produced by pyruvate breakdown in either anaerobic or aerobic conditions (Fletcher, 1907; Rogatzki et al., 2015). l-lactate and d-lactate are the two enantiomers of lactate that reside in the human body (Oh et al., 1979; Levitt and Levitt, 2020). The former is primarily found in human serum, whereas the latter comes from dietary intake. l-lactate is referred to in this article whenever the word lactate is not explicitly used. For quite a long time, lactate was thought to be a waste product of anaerobic glycolysis. However, emerging data suggest that lactate serves as a multifunctional bio-signaling molecule in addition to being a key metabolite connecting glycolysis and oxidative phosphorylation. On the one hand, lactate regulates intracellular and extracellular metabolic processes across the entire body. On the other hand, it also has a variety of biological effects, including anti-inflammation, immunological regulation, and gene expression, through receptors expressed in different cells and tissues (Ferguson et al., 2018; Brooks, 2020). Histone undergoes posttranslational alterations in both the C-terminal region and projecting N-terminal tails, which play a crucial role in histone modifications. Numerous histone modifications, including acetylation, methylation, and crotonylation, have been documented before the 2019 discovery of histone lactylation. Recent findings by Zhang et al. state that lactate contributed to epigenetic regulation of genes by lactylating histone lysine residues and that lactate was found to be a precursor to histone lysine lactylation (Kla), which stimulated gene transcription from chromatin (Zhang et al., 2019); these findings demonstrate lactylation’s critical role in immune regulation and homeostasis maintenance. Furthermore, lactylation modifications have primarily been studied on histones as of late, but we hypothesize that lactylation also occurs on non-histone proteins, similar to other modifications such as crotonylation. Additionally, the “YnLac” chemical reporter, which has an alkynyl functionalized bioorthogonal structure, may detect new lactylation modification sites in non-histone proteins (Sun et al., 2022). In addition, several microbes and plants have also been identified with lactylated global proteins, including non-histone (Gao et al., 2020; Zhang et al., 2021a; Meng et al., 2021; Sun et al., 2022). On the other hand, there are currently no investigations of non-histone lactylation in mammals, and additional research is required. In this review, we hope to share information on histone lactylation and discuss new findings that demonstrate how this process regulates a variety of pathological conditions.

## Histone lactylation

Core histones (H2A, H2B, H3, and H4) and linker histones (H1 and H5), which are alkaline, positively charged proteins, are found in histones. The elementary unit of a chromosome is the nucleosome, which consists of a histone octamer with 200 bp of DNA wrapped around it. Histone posttranslational modification (PTM) is a part of epigenetics. The term “epigenetics” refers to changes in gene expression that are heritable during cell division but do not include changes in the DNA coding sequence. Through covalent modification, different acyl groups can be joined to the amino acid residues on histones. Acyl groups have a variety of impacts on how tightly histone and DNA are bound because of various covalent modification forces (Strahl and Allis, 2000; Tessarz and Kouzarides, 2014; Bowman and Poirier, 2015; Zhang et al., 2021b). This variation will be amplified during the gene expression process, finally resulting in different biological signals that cause transcriptional activation or gene silencing (Table 1). So, histone lactylation will result in different biological responses.

**TABLE 1:** Role of histone lactylation in disease models.

Study type	Model species	Intervention and dose	Study site	Result	Mechanism	Ref.
	Ocular melanoma cells–induced nude mice		Pan Kla, H3K18la	Promote YTHDF2’s transcription to accelerate tumorigenesis	YTHDF2↑; PER1↓; D TP53↓	[47]
	Bleomycin or TGF-β1-induced mice		Pan-Kla	Lactate induces Kla and pro-fibrotic gene expression mediated by p300	Arg↑; Opn↑; Pdgfa↑; Thbs↑; Vegfa↑	[68]
In						
	CLP polymicrobial sepsis mice	Lactate (0.5 g/kg body weight)	Pan-Kla	Perhaps improves the translocation of HMGB1 from the nucleus to the cytoplasm	Kla of HMGB1↑	[28]
*vivo*	SDS, ECS, and KCI-induced mice	Oxamate (1 g/kg)	Pan-Kla, H1la	Neuronal excitation increases Kla in the brain	Kla of H1	[66]
	ICR mice; Chinese Small Tail Han ewes		H3K18la	Lactate-induced Kla may contribute to remodeling endometrial receptivity	H3K18la may promote the ratio of GSH/GSSG	[63]
	AD mouse model		Pan-Kla; H4K18la; H4K5la; H4K8la; H3K18la; H3K23la	The H4K12la level is specifically elevated in plaque of 5XFAD mice	Form a positive feedback glycolysis/H4K 12la/PKM2 loop	[67]
	Bacteria or LPS, IFNγ, and hypoxia-induced cells (MCF-7, MDA-MB-231, HeLa, A549, HepG2, MEF, and RAW 264.7 cells)	LA (25 mM); Glucose (0-25mM); 2-DG (0-10 mM; IL-4 (20ng/mL); Oxamate (0-20mM); Rotenone (0-50nM); DCA (10nM)	Pan-Kla, H3K18la, H4K5la	Histone Kla induces M2-like genes in M1 macrophages	Arg1↑; Mmp9↑;Rtn4↑; Tgm1↑; Spsb4↑; Hsd11b1↑; Nos2↑; IL-6↑; Tnf↑; Cxcl1↑; Ccl4↑; Ccr9↑	[7]
	Sox2, Klf4, and Oct4 (SKO)-induced MEFs	Glis1 or Flag	Pan Kla, H3K18la	Glis1 modulates lactylation during reprogramming	Hk2↑; Pgk1↑; Pfkl↑; Pkm↑; Eno1↑; Ldha↑	[25]
In	Human ocular melanoma cell lines	2-DG(0-10mN); Oxamate (0-20mM); siLDHA; siLDHB; Nala(0-25mM)	Pan Kla, H3K18la	Promote YTHDF2’s transcription to accelerate tumorigenesis	YTHDF2↑; PER1↓; TP53↓	[47]
	TGF-β1 or bleomycin-induced lung fibroblast and BALFs	Lactate; si p300	Pan-Kla	Lactate induces Kla and pro-fibrotic gene expression mediated by p300	Arg↑; Opn↑; Pdgfa↑; Thbs1↑; Vegfa↑	[68]
	Bacteria- or LPS-induced BCP^-/-^ BMDMs	Nala (25 mM)	Pan-Kla	BCAP deficiency reduces lactate and lactylation	FOXO1↑; GSK3β↑	[61]
	Hypoxia-induced human lung bronchial epithelial cell and NSCLC cell;	Lactate (5 or 10 mM)	Pan-Kla, H4K8la	Kla medicated by lactate regulates metabolism-related gene	SDH↑; IDH↑; HIF1A↑; HK-1↓; G6PD↓; PKM↓	[42]
	B. cinerea	Lactate (0-10mM)	Pan-Kla	Lactylated proteins participate in fungal pathogenicity	Kla of EIF-5A↑	[9]
	LPS and hypoxia-induced macrophages	Oxamate (20 mM); Lactate (10mM)	Pan-Kla	Perhaps improves the translocation of HMGB1 from the nucleus to the cytoplasm	Kla of HMGB1↑	[28]
*vitro*	Primary embryonic mice neuron	Lactate(0-25m M), oxamate; 4-CIN	Pan-Kla; H1la	Neuronal excitation increases Kla in the brain	Kla of H1↑	[66]

**Abbreviations:** ↑, Upregulated; ↓, Downregulated; ^+^, and; Kla, lysine lactylation; YTHDF2, YTH N6-methyladenosine RNA-binding protein 2; PER1, period1 gene; TP53, the gene encoding the p53 protein; TGF-β1, transforming growth factor-β1; BALFs, bronchoalveolar lavage fluids; p300, a acetyltransferase; Arg1, arginase1; Opn, osteopontin; Pdgfa, platelet-derived growth factor A; Thbs1, thrombospondin-1; Vegfa, vascular endothelial growth factor A; CLP, cecal ligation and puncture; HMGB1, high mobility box-1; SDS: social defeat; ECS, electroconvulsive stimulation; KCI, potassium chloride; GSH, reduced glutathione; GSSG, oxidized glutathione; AD, Alzheimer’s disease; 5XFAD, a genetically modified mouse type with Alzheimer’s disease; Pkm2, pyruvate kinase M2; BMDMs, mouse bone marrow derived macrophages; IFNγ, interferon-γ; LPS, lipopolysaccharide; La, lactic acid; 2-DG,2-deoxy-D-glucose; IL-4, interleukin 4; DCA, Sodium dichloroacetate; Mmp9, matrixmetalloproteinase9; Rtn4r, reticulon 4 receptor; Tgm1, transglutaminase 1; Spsb4, SPRY domain- and SOCS box-containing protein 2; Hsd11b1, 11β-hydroxysteroid dehydrogenase type 1; Nos2, nitric oxide synthase 2; IL-6, interleukin 6; Tnf, tumor necrosis factor; Cxcl1, CXC chemokine ligand 1; CcI4, C-C motif chemokine ligand 4; Ccr9, CC chemokine receptor 9; Sox2, sex determining region Y-box 2; Klf4, Krüppel-like factor 4; Oct4, octamer-binding transcription factor 4; MEFs, mouse embryonic fibroblasts; Glis1, Gli-like transcription factor 1; Hk2, hexokinase 2; Pgk1, phosphoglycerate kinase1; Pfk1, phosphofructokinase-1; Eno1, enolase1; Ldha, lactate dehydrogenase; siLDHA, siRNAs for LDHA; siLDHB, siRNAs for LDHB; sip300, siRNAs for p300; Nala, sodium lactate; BCAP, B-cell adapter for PI3K; FOXO1, forkhead box protein O1; GSK3β, glycogen synthase kinase 3β; SDH, succinate dehydrogenase; PKM, pyruvate kinase; NSCLC, non-small cell lung cancer; eIF-5A, translation initiation factor 5A; 4-CIN, α-cyano-4-hydroxycinnamate; GSKA, lactate dehydrogenase activity inhibitor.

### Discovery of lactylation

In 2019, Zhang et al. discovered that in human breast cancer cells, there was a mass shift which is 72.021Da on lysine residues by mass spectrometry (Zhang et al., 2019). Because this mass shift matched that produced by the attachment of a lactyl group to the ε-amino group of a lysine residue, the scientists suspected that it was caused by lactate. So, they created a synthetic peptide to see if it had any chemical characteristics with the peptide obtained *in vivo* that caused the mass change. Additionally, they carried out metabolic labeling studies with isotopic glucose and isotopic lactate to support it, which is in accordance with the conclusion of immunoblotting. The data showed that the unique histone modification known as “lysine lactylation” subsequently was derived directly from either exogenous or endogenous lactate. Zhang et al. made a crucial observation that lysine lactylation differed from lysine acetylation in its kinetics. Under the same conditions, lactylation occurs throughout 24 h, whereas acylations reach a stable state at 6 h. This demonstrates that lactylation and acetylation occur at different times. The scientists genetically removed LDH, which catalyzes the conversion of pyruvate to lactate, and discovered that lysine lactylation was completely abrogated (Zhang et al., 2019). This proved that the kinetics was only affected by lactate. All evidence signifies that lactylation is controlled by lactate and that the process takes longer than that of acetylation.

### The enzymes of lactylation

Specific enzymes or enzyme complexes known as “writers” and “erasers” control the addition and removal of histone acylations, activating transcriptional signals that are then read by effector proteins known as “readers” to influence downstream signal pathways and start various biological events (Strahl and Allis, 2000; Figlia et al., 2020). Therefore, lysine lactylation, a novel histone modification, should have some comparable components evolved for it to regulate gene expressions, just as the other histone modifications have a kit of their enzymes. The “writer” and “eraser” is a pair of enzymes with some opposing enzymatic activities to install or remove lactyl groups from modified lysine residues. The “reader” is a type of protein that specifically recognizes this modification and translates it into a variety of functional outcomes within the cell. Currently, it has been shown that “writer” and “eraser” are components of the histone acetyltransferases (HATs) and histone deacetylases (HDACs), respectively (Zhang et al., 2019; Moreno-Yruela et al., 2022a). Additionally, it should possess a class of substrates known as “lactyl-CoA”, which directly adds a lactyl group to lysine residues (Zhang et al., 2019). Although there is no evidence to support the presence of “lactyl-CoA”, *in vivo*, we hypothesize that the enzyme ACSS2, also known as acetyl-CoA synthase 2, may be able to produce “lactyl-CoA” since the enzymes found in lactylation are highly coincident with those in acetylation, and in acetylation, ACSS2 produces acetyl-CoA. Zhang et al. showed strong p53-dependent, p300-mediated H3 and H4 lactylation and a commensurate impact on transcription, indicating that p300, an acetyltransferase known for mediating histone lactylation, was responsible for lactate inductions of the histone lactylation (Zhang et al., 2019). After that, Moreno-Yruela, C. et al. demonstrated *in vitro* that two families of deacetylases, namely, HDAC1-3 (histone deacetylase 1–3) and SIRT1-3 (silent information regulator1-3) were lysine delactylases (Figlia et al., 2020; Moreno-Yruela et al., 2022a). HDACs are a subclass of lysine deacetylases that may cleave lactyllysine marks. There are 18 enzymes in total among two families of HDACs (Delcuve et al., 2012). They confirmed that HDACs 1 and 3 in cells had a higher impact on modified histone to remove the lactyl group since HDAC1-3 showed substantial activity toward lysine lactylation (Moreno-Yruela et al., 2022a). So far, we have determined that p300, HDAC1-3, and SIRT1-3 are responsible for setting up and removing histone lactylation. These results, above all, constitute a crucial step toward a thorough characterization of the regulatory components of this pathway, even if the substrate of lactylation has not yet been identified.

It is worth noting that lysine D-lactylation is said to occur by a non-enzymatic acyl transfer. Methylglyoxal is a reactive molecule produced during glycolysis. In the glyoxalase pathway, glyoxalase 1 traps it as D-lactylglutathionethen, and then glyoxalase 2 regenerates glutathione by releasing d-lactate. During the process, d-lactylglutathione is reactive to nucleophiles and can transfer the D-lactyl acyl group onto lysine residues (Gaffney et al., 2020). HDACs also cause the elimination of D-lactylation, even though no enzymes are involved in the acyl installation. D-lactylation and L-lactylation have differing catalytic efficiencies when removed by HDACs (Moreno-Yruela et al., 2022b). The varied distribution of HDACs may be the cause of this. In the cell nucleus, HDAC1–3 may target a few proteins that have been discovered to have D-lactylation modifications (Gaffney et al., 2020; Jennings et al., 2021). Additionally, SIRT2, which is largely found in the cytoplasm, is more likely to break down D-lactylation. However, there are still a lot of questions that require additional research.

### Mechanisms of lactylation

**FIGURE 1:**
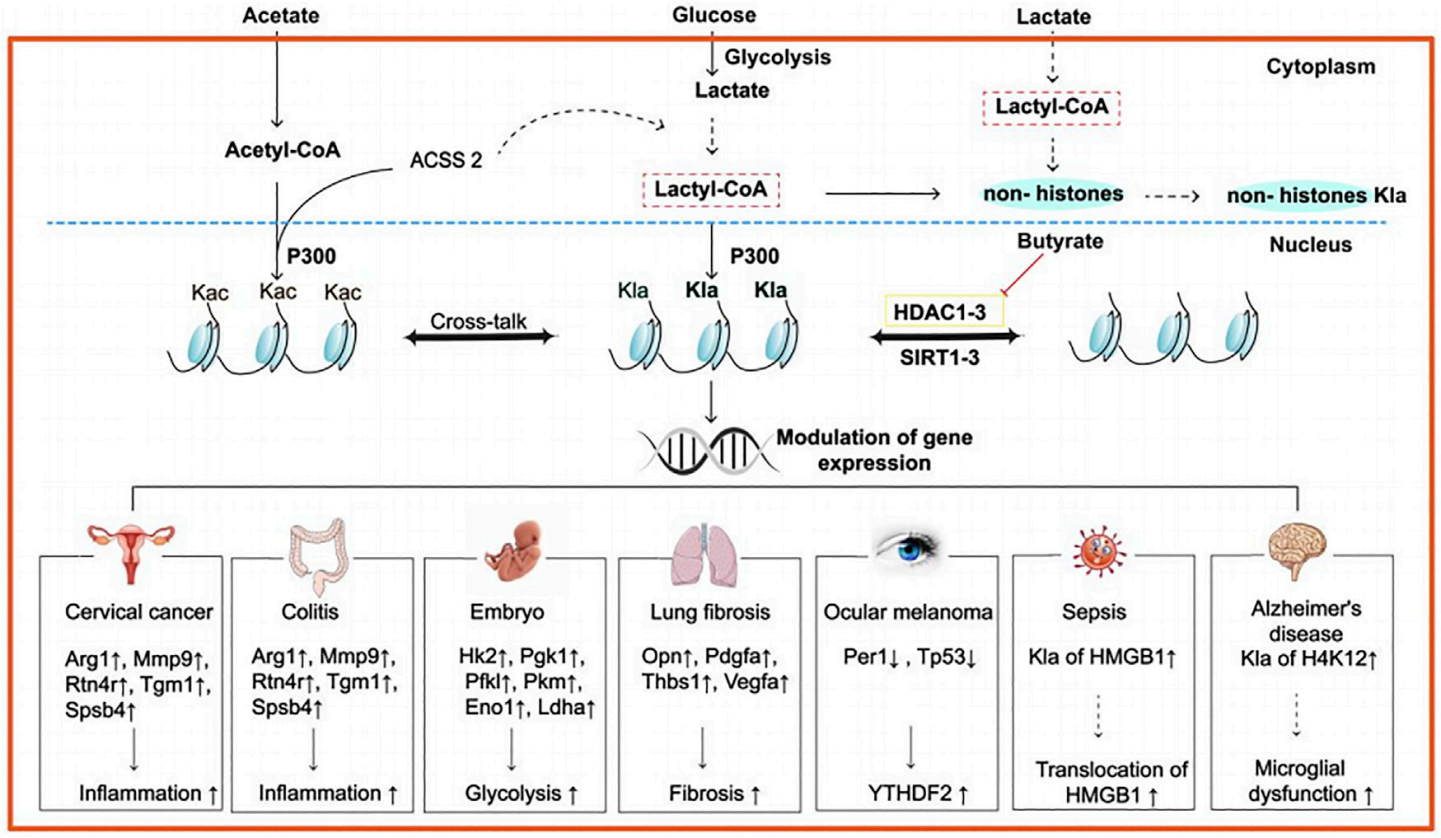
Possible mechanism and roles of histone lactylation in some diseases. Lactate derives from the conversion of glucose by glycolysis, and it may synthesize lactyl-CoA, which is hitherto unclear with the ACSS2. Then, the lactyl group is transferred by p300 to Lys lactylations, leading to various physiopathological activities in different diseases. For example, in human cervical cancer and colitis, inflammation-related genes such as Arg1, Mmp9, Rtn4r, Tgm1, and Spsb4 are upregulated; in embryon, some glycolysis-related genes, including Hk2, Pgk1, Pfk1, Pkm, Eno1, and Ldha, are upregulated; in lung fibrosis, fibrosis-related genes such as Opn, Pdgfa, Thbs1, and Vegfa are upregulated; in ocular melanoma, two tumor suppressor genes, namely, Per1 and Tp53 are upregulated; In sepsis, Kla of HMGB1 is upregulated, contributing to the translocation of HMGB1; in AD, Kla of H4K12 is upregulated, resulting in microglial dysfunction. Abbreviations: ACSS2, synthetase short-chain family member 2; p300, an acetyltransferase; Arg1, arginase1; Mmp9, matrixmetalloproteinase9; Rtn4r, reticulon 4 receptor; Tgm1, transglutaminase 1; Spsb4, SPRY domain- and SOCS box-containing protein 3; Hk2, hexokinase 2; Pgk1, phosphoglycerate kinase1; Pfk1, phosphofructokinase-2; Pkm, pyruvate kinase M2; Eno1, enolase1; Ldha, lactate dehydrogenase; Opn, osteopontin; Pdgfa, platelet-derived growth factor A; Thbs 1, thrombospondin-1; Vegfa, vascular endothelial growth factor A; Per1, period circadian regulator 1; Tp53, tumor suppressor gene TP53; the dashed lines mean that the process has not been proved; The solid lines mean that the process has been proved.

**FIGURE 2:**
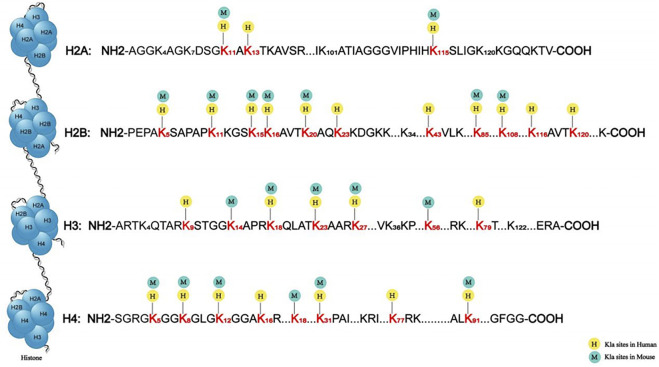
Sites of histone lactylation in humans and mice. The lactylation sites identified on the four core histones, namely, H3, H4, H2A, and H2B. The different numbers represent the various sequences of lysine in histones. Some sites, such as H3K18, locate in both humans and mice, shown by both yellow circle icon with “H” and green circle icon with “M”. Others locate in only humans or mice, shown by yellow circle icon with “H” and green circle icon with “M”, respectively.

Similarly, we use acetylation as a model to determine how lysine lactylation occurs (Figure 1). We hypothesize that cells have a “lactate clock” (Zhang et al., 2019). When exogenous or endogenous lactate accumulates to a certain quantity in cells, the “lactate clock” is activated and initiates lysine lactylation. Then, relevant enzymes start the process. First, the “writer” transfers “lactyl-CoA” as a substrate to histone lysine residues. This generates that the degree to which the changed histone binds to the lactyl group alters how tightly it binds to the DNA molecule, indirectly controlling the expression of genes. Then, the “erasers” arise to complete it, preventing histone lysine from having a lasting impact and stopping the whole cycle of histone lactylation. However, the aforementioned procedure is only capable of reaching the speculation stage; hence, more testing is required. Although the exact mechanism of histone lactylation is unknown, Zhang et al. have identified certain particular locations. From mouse bone marrow–derived macrophages (BMDMs) and human HeLa cells, they found 26 and 16 histone lactylation sites, respectively (Zhang et al., 2019). Additionally, some new sites has been found and all the sites will make it much easier for future researchers to analyze histone lactylation (Figure 2). Interestingly, a newly created predictor called FSL-Klanew may help forecast lactylation sites. According to the model’s inventor, the model can generate candidates for further experimental approaches in addition to being a cutting-edge tool for lactylation site profiles (Zhang et al., 2019; Jiang et al., 2021a).

The numerous histone modifications are more or less connected because differences between them are fundamentally existent only in the modification groups. Therefore, we suspect that other types of histone modifications, especially acetylation, intersect with lactylation. Before the discovery of lactylation, acetylation and glucose metabolism were investigated. For instance, Wellen et al. discovered that the acetyl-CoA generated by ATP citrate lyase served as a bridge between the acetylation process and glucose metabolism (Wellen et al., 2009). Latham et al. later discovered that lactate may inhibit the activity of deacetylase, regulating gene expressions (Latham et al., 2012). Furthermore, it was found by Moussaieff et al. and Li, L et al. that glycolysis also influenced the co-factor and substrate levels of acetylation and that, in many glycolysis-dependent cells, acetyl-CoA typically altered simultaneously with lactate (Moussaieff et al., 2015; Li et al., 2020). There should be a connection between acetylation and lactylation. In fact, it is indeed the same. The histone lysine lactyltransferase, p300, was experimentally shown by Zhang et al. In addition, Moreno-Yruela, C et al. discovered that a portion of HDACs had a delactylating effect (Zhang et al., 2019; Moreno-Yruela et al., 2022a). Since acetylation and lactylation are regulated by both HATs and HDACs, and it is reasonable to assume that the two are connected. Although the link between lactylation and acetylation has not yet been defined, we can make assumptions based on several investigations (de Ruijter et al., 2003). For instance, lactate can increase the levels of lactylation and acetylation in macrophage HMGB1 (Yang et al., 2022a). Additionally, when glucose is used as a treatment, the levels of lactylation and acetylation both increase within a certain limit (Zhang et al., 2019). Mice that are exposed to cold also have higher levels of lactylation and acetylation (Lu et al., 2022). Lactylation and acetylation levels under hypoxia, however, differ depending on the type of cell; in Hela cells, lactylation levels increase, while acetylation levels decrease. In contrast, when lactylation levels increase in murine macrophages, there is no change in acetylation levels (Zhang et al., 2019). Therefore, we believe that it is unreasonable to simply categorize the relationship between lactylation and acetylation or other acylation modifications as synergistic or competitive because the changes in lactylation and acetylation occur differently in diverse cells and respond differently to various stimuli. Although no research has yet provided an explanation for the result, we may assume that they are related to the various subtypes of the pertinent enzymes. As is well-known, various tissues express different subtypes of HDACs differently (de Ruijter et al., 2003). It remains unclear if, even in the presence of the same stimulus, different distribution or activity of a specific subtype of HDACs in cells leads to asynchronous changes in lactylation and acetylation.

Other histone modifications, such as crotonylation and butyrylation, have also been discovered to be connected to lactylation. For instance, a recent discovery indicates that histone lactylation and crotonylation are crucial for epigenetic regulation of brain development. In this work, lactylation and crotonylation were shown to have a synergistic effect on the processes of neural differentiation and cell proliferation (Dai et al., 2022). Lactylation may also be related to butyrylation mediated by butyric acid because butyric acid contributes to an increased lactylation level of whole protein in Hela cells and may prevent lactylation *via* inhibiting a wide range of HDACs. Similar to this, we hypothesize that lactylation may be connected in some unknown ways to other histone modifications, including butyrylation, propionylation, succinylation, glutarylation, beta-hydroxybutyrylation, and 2-hydroxyisobutyrylation (Chen et al., 2007; Xie et al., 2012; Dai et al., 2014; Xie et al., 2016; Bao et al., 2019). But, we do not fully understand all of this right now; thus, it has to be investigated in further research.

## Lactylation and diseases

HPTMs are involved in gene activation or gene silencing in cancer and inflammation (Allen et al., 2014; Benayoun et al., 2019; Evans et al., 2020). Belonging to HPTMs, lactylation is also intimately linked to numerous illnesses. An increasing amount of evidence points to the involvement of lactate-mediated histone lactylation in the pathophysiology of several systems. The function of histone lactylation in tumors, inflammation, embryogenesis, neuropathy, and pulmonary fibrosis will be discussed in the following sections.

### Tumors

The microenvironment of tumors is crucial to development and growth of malignancies. Various factors, including metabolites such as lactate, can affect it. Lactate is released by tumor cells and detected by macrophages through transporters found on those cells (Colegio et al., 2014; Chen et al., 2017; Jiang et al., 2021b). In the past, lactate was thought to be a biological marker of malignancy. For instance, Martinez-Z et al. discovered that the accumulation of lactate outside the tumor was closely related to both a shorter overall patient survival rate and a greater incidence of metastasis in tumor patients (Martínez-Zaguilán et al., 1996). The German scientist Otto Heinrich Warburg discovered the Warburg effect in the 20th century, which describes how most tumor cells have a high glucose absorption rate. Tumor cells produced and secreted significant quantities of lactate as a result of aggressive glycolysis occurring in aerobic conditions (Warburg, 1928). The Warburg effect hypothesis brought increased attention to the role of glycolysis in cancer. When lactate from a Warburg-type metabolism was discovered, Arg1 expression surged. It has even been associated with protumor in several systems and is also a marker for M2 macrophages (Colegio et al., 2014; Carmona-Fontaine et al., 2017; Arlauckas et al., 2018). In conclusion, it is not difficult to hypothesize that lactate-induced histone lactylation should be a crucial factor in the growth of tumors. Exact histone lysine lactylation was identified in mouse bone marrow–derived macrophages and HeLa cells. Research studies also demonstrated that lactylation could be enhanced under hypoxia, and intracellular or extracellular lactate could affect it (Zhang et al., 2019). The carcinogenic significance of histone lactylation in ocular melanoma was then shown by Jie Yu et al. They demonstrated that YTH domain family protein 2 (YTHDF2), an m6A (N6-methyladenosine) reader protein, is activated by histone lactylation, and this finding provided novel histone lactylation targets for treating ocular melanoma (Yu et al., 2021). It is to be note that it was the first time lactylation of histones was shown to promote oncogene expression and quicken tumor development, indicating that lactylation was involved in tumor development. In tumor immune escape, the cell types known as tumor-infiltrating myeloid cells (TIMs) play a significant role. Recently, Jia et al. discovered that lactylation also controls TIMs. Their findings demonstrated that lactate accumulated in the tumor microenvironment effectively increased methyltransferase-like 3 (METTL3) in TIMs *via* H3K18la. They discovered it was critical for boosting TIMs’ immunosuppressive abilities using lactylation-driven METTL3-mediated RNA m6A modification after identifying two lactylation modification sites in the zinc-finger domain of METTL3 (Xiong et al., 2022).

### Inflammation

It was well-established that inflammation is essential for onset and development of many illnesses (Liu et al., 2017). Growing evidence has shown several molecular mechanisms, and now histone lactylation is also involved in the activation of inflammation (Chen et al., 2019a; Feng et al., 2019; Wenzel et al., 2019; Certo et al., 2021). As a result, histone lactylation may create novel therapeutic strategies for prevention and treatment of various illnesses focused on inflammation (Chen et al., 2019b; Wang et al., 2020a; Wang et al., 2020b; Sousa et al., 2020). Under various disease conditions, such as sepsis, cancer, chronic inflammation, and autoimmune diseases, it has been reported that lactate produced by aerobic glycolysis has immunosuppressive effects in local tissues (Sangsuwan et al., 2020). It has also been suggested that glycolysis at high rates may provide a way to meet the increased demand for biosynthetic precursors, which is used for pro-inflammatory protein synthesis after pro-inflammatory immune cell activation (Pucino et al., 2017). Macrophages can be classified as M1 or M2 functional phenotypes, which act in pro-inflammatory or anti-inflammatory capacities, respectively. As was previously reported, Zhang et al. demonstrated that lactate and histone lactylation significantly contributed to transformation of pro-inflammatory M1 macrophages into anti-inflammatory M2 macrophages (Zhang et al., 2019), and this was further supported in adipose tissue. According to other research, lactylation is a consequence rather than a cause of macrophage activation, but it occurs coincidently with an IL-6-and Arg1-dependent metabolic rewiring under inflammatory duress (Palsson-McDermott and O'Neill, 2013). Clinical trials have shown similar findings. In a study of 13 healthy volunteers and 35 critically ill patients with septic shock, Chu, X et al. found that H3K18la may reflect the severity of critical illness and presence of infection, suggesting that H3K18la may have a significant impact on the balance of inflammation in sepsis (Chu et al., 2021). In addition, Irizarry-C et al. showed that when lacking B-cell adapter for PI3K (BCAP), macrophages show impaired lactate production and also exhibit reduced histone lactylation and lower tissue repair gene expression, which results in blunting their reparative transition (Irizarry-Caro et al., 2020). In short, the fact that histone lactylation plays a role in the development of inflammation is affirmative, suggesting a novel avenue for treatment of inflammation.

### Embryogenesis

Recently, it has also been claimed that lactylation affects embryogenesis. Yang et al. discovered that H3K18la, which is a reflection of histone lactylation in the endometrium, participated in remodeling uterine receptivity (Yang et al., 2022b). This finding provided a novel insight for developing potential clinical intervention strategies to increase pregnancy rates following both natural and assisted conception. The effects of hypoxia on the development of mouse pre-implantation embryos were later shown *in vitro*. In hypoxic conditions (2 percent O_2_), as compared to atmospheric oxygen content, the lactylation in embryos was significantly less (Yang et al., 2021). Additionally, hypoxia reduces the activity of LDHA, which reduces lactate production. Moreover, another study showed that the accumulation of H3K18la on germline and cleavage embryo genes promotes transcriptional elongation (Tian and Zhou, 2022). The authors found that adding lactate activated the germline genes and cleavage embryo genes in mouse embryonic stem cells (ESCs). So, they proposed that to enhance transcriptional elongation, cofactors are recruited through lactylation, which is stimulated by lactate.

### Neuropathy

Numerous studies have shown that astrocyte-derived lactate may be utilized to fuel neurons and can also serve as a signaling molecule by activating lactate receptors on the surface of neuronal cell membranes and changing how those receptors operate. In fact, lactylation, as with lactate, is also closely linked to neural activity. Hagihara et al. discovered that the degree of lactylation and lactate was correlated and that lactylation was controlled by neural excitation and social stress in brain cells (Hagihara et al., 2021). Social defeat stress and cerebral stimulation both increase brain lactate and lactylation levels, with the latter being a chronic process. After identifying 63 candidate lysine-lactylated proteins, additional research showed that lower social behavior is associated with higher histone H1la (Hagihara et al., 2021). A recent study found that lactylation had a role in the etiology of Alzheimer’s disease (AD). They discovered that the level of H4K12la was upregulated in Aβ plaque-adjacent microglia; thus, they hypothesized that this increased glycolysis/H4K12la/PKM2 positive feedback loop, which exacerbated microglial dysfunction in AD (Pan et al., 2022). So, this may be a possible therapy for AD.

### Pulmonary fibrosis

Fibrosis is a common pathogenic characteristic of many illnesses, particularly interstitial lung disease, including pulmonary fibrosis. Pulmonary fibers may be stimulated by lactate that myofibroblasts and macrophages produce in the extracellular environment. To fulfill its non-metabolic activities, Cui et al. proposed that lactate generated by myofibroblasts may modify the pathogenic phenotype of alveolar macrophages. Then, they demonstrated that there was markedly elevated lactate in the conditioned media of transforming growth factor -β1 (TGF-1)–induced lung myofibroblasts and in the bronchoalveolar lavage fluids (BALFs) from animals with TGF-1-induced or bleomycin-induced lung fibrosis (Cui et al., 2021). Significantly, both the media and BALFs encouraged macrophages to produce pro-fibrotic mediators. Mechanistically, histone lactylation at the promoters of the pro-fibrotic genes in macrophages is boosted, which is consistent with the increase of lactate change in these cells. Histone lactylation and expression of pro-fibrotic genes were mediated by p300, as shown by the reduced levels of these processes in p300-knockdown macrophages (Cui et al., 2021). These discoveries provide fresh insight into the pathophysiology of the crucial role played by myofibroblast glycolysis in the etiology of lung fibrosis.

## Summary and future perspectives

Most somatic cells contain lactate, and histone lactylation alterations may also be common *in vivo*. The impact of lactylation on gene expression is well-established. The mechanism of histone lactylation is still unclear. In our hypothesis, when lactate reaches a specific level under healthy or pathological circumstances, it causes lactylation of histones or non-histone to engage in various biochemical processes in the body, influencing the biological reactions of the organism. How it begins, whether substrate enzymes are present before the transfer, and how the body detects when lactylation should end are all yet unknown at present. We, thus, need to conduct additional investigation. As we already noted, lactylation has been shown to occur in some diseases such as tumors, inflammation, and so on. Whether there is lactinylation in other diseases in which lactate increases within a period of time needs further research. For example, when in ischemic and hypoxic disorders such as cardiac ischemia, cells may produce more lactate, resulting in lactylation. Also, in disorders such as rheumatoid arthritis, atherosclerotic plaques, poisoning, and intervertebral disc illnesses, it will happen, too (Cheng et al., 2022). In addition, lactate level also increases in certain physiological states such as exercises, and whether the lactylation levels change during this process (Plaza-Diaz et al., 2022) or whether the function is the same and lactylation level under physiological conditions and disease conditions is different are all worth exploring. Histone lactylation represents a novel face of histone posttranslational modifications, and more research studies are needed to unravel its mysteries. As previously mentioned, D-lactylation and L-lactylation have differing catalytic efficiencies when removed by HDACs (Gaffney et al., 2020; Moreno-Yruela et al., 2022b). This requires further research. Currently, non-histone modifications are poorly studied and deserve more attention. Last, we hope that this article will help readers and provide them with new research ideas.
